# Modalities of using endodontic nickel-titanium rotary instruments and factors influencing their implementation in dental practice

**DOI:** 10.1186/s12903-018-0660-x

**Published:** 2018-11-21

**Authors:** Ahmad A. Madarati, Adnan A. Habib

**Affiliations:** 1 Restorative Dental Sciences Department, College of Dentistry, Taibah University, P.O Box 2898, Madina, 43353 Saudi Arabia; 2 Restorative Dentistry Department, Faculty of Dentistry, Aleppo University, Aleppo, Syria; 3 Al-Farabi College of Dentistry, Riyadh, Saudi Arabia

**Keywords:** NiTi, Rotary files, Endodontics, Modalities, Adoption, Questionnaire, Uptake, Survey, Glide path, Root canal

## Abstract

**Background:**

To establish the extent of using nickel titanium rotary instruments (NiTi-RIs), to identify reasons for using / not using NiTi-RIs, to explore usage modalities and to identify factors and measures that can increase implementation of NiTi-RIs in general dental practice.

**Methods:**

Two pilot questionnaires were conducted on academic staff members at College of Dentistry, Taibah University, general dentists (GDs) and endodontists to finalise the questionnaire. A sample size was calculated considering the expected and minimum accepted response rates (60 and 48%, respectively) and a 99.9% Confidence Level. The online-questionnaire was sent to 600 GDs and all endodontists (175) working in Saudi Arabia. A reminder was emailed after 10 weeks to encourage non-respondents to complete the questionnaire. Responses, were collected and converted into numerical data which were analysed using the Chi-square test *(p* = 0.05*)*.

**Results:**

Significantly most respondents (71.9%) used NiTi-RIs *(p < 0.001)*; with more endodontists (96.9%) than GDs (60%). Most users (62.5%) had been using NiTi-RIs for *More than 3 years (p < 0001)*. The trend of using NiTi-RIs increased as *participants’ experience* and the number of *root-canal treatments performed per week* increased *(p = 0.021)*. While most respondents (45.3%) used NiTi-RIs because of *faster root-canal preparation*, the majority of non-users (85.3%) didn’t do so because of *high cost*. The highest proportion (43.3%) reported *better undergraduate education* as the most important factor that can significantly increase NiTi-RIs usage. The majority (91.8%) prepared *glide-path* before using NiTi-RIs; especially with *stainless steel hand-files* (63.3%).

**Conclusions:**

NiTi-RIs are relatively well adopted in Saudi dental practice. However, *better education*, especially during undergraduate training and *lower cost* can increase their usage. Overall, clinicians showed good awareness of NiTi-RIs usage aspects which reflected on usage modalities.

## Background

Proper debridement of the root-canal system by cleaning and shaping is an essential procedure for successful root-canal treatments (RCTs) [1]. Developing a continuously tapered funnel as well as maintaining the original shape of root-canals and the apical foramen in its original position are main objectives of good cleaning and shaping [2]. Therefore, knowledge and understanding of techniques and instruments used for cleaning and shaping are paramount. While root-canal instruments had been first made of carbon steel, stainless steel instruments (SSIs) were dominant for few decades due to their greater ductility, which allowed greater resistance to fracture [3]. However, these instruments lack flexibility and cannot keep the original root-canals’ shape [4, 5]. They also may lead to mishaps and complications that may hinder cleaning and shaping of root-canal systems [6].

In the early 1960s, the nickel-titanium (NiTi) alloy was developed by WF Buehler and then was introduced into dentistry [7]. As a super-elastic alloy, deformations of as much as 8% strain can be fully recoverable compared to less than 1% with stainless steel [4, 7]. Also, and unlike SSIs, which are manufactured by twisting, NiTi instruments are machined, which allows producing various instruments designs [7]. This with the super-elasticity property are almost the main reasons for using the NiTi alloy widely in manufacturing endodontic instruments; especially rotary instruments (RIs).

Many studies have reported better performance of NiTi-RIs, in cleaning and shaping of the root-canal system, compared to SSIs [6, 8–10]. A 1-year recall study comprising 40 cases, reported a significantly better success rate for teeth prepared with NiTi hand files than with SS K-files [6]. A very recent study investigated factors affecting the status of periapical tissues of endodontically treated teeth among German urban populations [10]. It has found that teeth instrumented with NiTi-RIs had a significantly better outcome when compared to those performed with SSIs [10]. Unsurprisingly, NiTi instruments were recommended for safer and faster instrumentation of root-canal systems. Several studies have shown different trends in implementing NiTi instruments, especially RIs, in endodontic and general dental practice of different countries. Barbakow & Lutz found that 80% of Swiss dentists used NiTi-RIs in their practice [11]. Parashos & Messer found that only 22% of general dentists (GDs) and 64% of endodontists, in Australia, were using NiTi-RIs [12]. Another questionnaire showed that 93% of endodontists and 65% of GDs in United Kingdom were using NiTi-RIs [13]. A relatively recent study found that 74% of GDs in the United States were using NiTi-RIs [14]. More importantly, one systematic review confirmed the differences in daily general practice and academic teaching among different countries [15]. Nevertheless, there is need for more information regarding the different usage modalities and reasons for using/not using NiTi-RIs [12]. This is especially important as significant improvement in RIs designs and properties has been witnessed in the last decade [16]. None of previous studies investigated some important aspects such as: preparing glide-path before using NiTi-RIs and dentists’ beliefs about measures that contribute into better implementation of NiTi-RIs in general dental practice. Such information can be obtained mainly by questionnaires studies [13, 17], especially if they are well planned and conducted; so that results are representative and can be generalised [17, 18].

Consequently, the aims of this questionnaire study were to establish the extent of using NiTi-RIs in Saudi dental practice, to explore usage modalities and to identify factors that may contribute to better implementation of NiTi-RIs in Saudi general dental practice.

## Methods

An official ethical clearances was obtained from the Research Ethics Committee (REC) at College of Dentistry, Taibah University. The study was conducted without the need for participants’ consent form and in accordance to the World Medical Association’s Helsinki Declaration. A first pilot online questionnaire was distributed to academic staff members at the College of Dentistry, Taibah University to ensure that the questions are easily understood. A second pilot study was conducted on 30 GDs and 10 endodontists to finalise the questionnaire. The final online questionnaire compromised questions that covers the following three main aspects:

A. **Demographic & General Information:** category of participants (GDs, endodontists, others), participants' experience, types of practice (government and private), number of RCTs performed per week.
B. **Using of NiTi-RIs;** experiences period in using NiTi-RIs and reasons for using/not using them.
C. **Modalities of NiTi-RIs Usage:** glide-path preparation before using NiTi-RIs, preparation sequences, using of the hybrid technique, and NiTi-RIs usage according to teeth types & canals’ geometries:

A sample size was calculated using the STATCLAC programme considering the population size (total of GDs in Saudi Arabia) (7050) and the expected and minimum accepted response rate; 60 and 48%, respectively. A sample size of 239 GDs would have given a 99.9% confidence level. However, it was determined to send the questionnaire to 600 GDs for a better statistical analysis in subgroups and to minimize the number of expected cells that count less than five in cross-tabs tables. The sample of GDs was selected randomly using the systematic sampling method [12, 13]. Considering that the 600 were to be selected from the 7050 GDs listed in the Saudi Dental Register, an interval between two selected GDs would be 11. After determining a random number from the Dental Register list, the 11^th^ following listing dentist in the dental registered was selected. If the 11^th^ following listing was not a dentist, the next GD was selected. The process continued till the 600 study sample was completed. The final questionnaire was sent electronically using the Google-Drive tool (http://www.google.co.uk) to the 600 selected GDs and all endodontists (175) working in Saudi Arabia. The email explained the study’s aims and indicated that the study would be conducted in a way that keeps participants’ identities anonymous. A further email was sent after 10 weeks to remind those who did not respond to the first sent-out questionnaire, so that they can complete the questionnaire. Responses, as an excel sheet, were collected and converted into numerical data that were inserted into SPSS 20 for Windows software (SPSS Inc., Chicago, USA). Data were analysed using the Chi-square test at *p = 0.05.*

## Results

### Response rate & participants’ classification

Of the 395 who responded to the questionnaire (out of 775 recipients), 264 (66.8%) were GDs, 97 (24.6%) were endodontists, 12 (3%) were students or residents in endodontic postgraduate programmes, and 22 (5.6%) were others. Since the use of NiTi-RIs is included in the curriculum of endodontic postgraduate programmes in Saudi Arabia, enrolled postgraduate students or residents were classified as endodontists. Thirty-two respondents (24 GDs, 7 others and one endodontist) were excluded as they never performed RCTs. Consequently, the final response rates were as follow:

- Overall: 395/743 (775–24) = 53.16%
- Non-endodontists response: 298/569: 52.37%
- Endodontists response rate: 97/174 = 55.74%.

Overall, most respondents (71.9%) used NiTi-RIs (*p < 0.001);* with a significantly more endodontists (96.9%) than GDs (60%) *[p < 0.001]* (Table 1)*.* Whilst the highest proportion of GDs (29.5%) had *up to 3 years*’ experience, the highest proportion of endodontists (47.4%) had > *15 years’ experience* (*p < 0.001*). The trend of *using* NiTi-RIs significantly increased as *participants’ experience* increased *(p = 0.001*). Whilst 47.8% used NiTi-RIs within the *Up to 3 years’ experience* group*, NiTi-RIs users* significantly increased to *85.6%* within the *7.1 to 15 Years’ experience* group (*p < 0.001).*

**Table 1:** Usage of NiTi-RIs acording to Participants' Classifications and Experiences

Respondents’ Classification	Experience of Respondents (Years)				
	Up to 3	3.1 to 7	7.1 to 15	More than 15	Total
General Dentists	29.5 (20.8)	27.3 (29.2)	22.7 (31.2)	20.5 (18.8)	100 [144] (60)
Endodontist	0 (0)	15.5 (16.1)	37.1 (38.7)	47.4 (45.2)	100 [93] (96.9)
Endo Postgraduates	25 (25)	50 (50)	25 (25)	0 (0)	100 [12] (100)
Other	4.5 (0)	31.8 (33.3)	36.4 (41.7)	27.3 (25)	100 [15] (80)
Total	20.8 (47.8)	25.3 (75.3)	27.1 (85.6)	26.8 (71.9)	100 [261] (71.9)

The values in brackets and parentheses represent the number and proportion, respectively, of respondents who used NiTi-RIs

Overall, there were significant differences between GDs and endodontists regarding the number of RCTs performed per week*.* Whilst only 6.8% of GDs performed *more than 12 cases*, 33.9% of endodontists did so (*p < 0.001)* (Table 2). The trend of *using NiTi-RIs* significantly increased as the *number of weekly performed RCTs* increased (*p = 0.021*)*.*

**Table 2:** Number of weekly performed RCTs & work’s sector

Respondents’ Classification	Number of RCT cases performed per week					
	Never Do RCTs	1–3	4–6	7–12	More than 12	Total
General Dentists	9.1	21.6 (22.9)	30.7 (33.3)	31.8 (37.5)	6.8 (6.2)	100 (60)
Endodontist	0.9	8.3 (8.6)	14.7 (15.2)	42.2 (43.8)	33.9 (32.4)	100 (96.9)
Other	31.8	18.2 (25)	22.7 (33.3)	13.6 (25)	13.6 (16.7)	100 (80)
Total	8.1	17.7 (64.3)	25.8 (66.7)	33.7 (77.4)	14.7 (77.6)	100 (71.9)
Respondents’ Experience (Years)	Number of RCT cases performed per week					
	1–3 cases	4–6 cases	7 to 12 cases	More than 12 cases	Total	
Up to 3	30.4	50.7	18.8	0	100	
3.1 to 7	18	41.6	33.7	6.7	100	
7.1 to 15	17.3	13.5	48.1	21.2	100	
More than 15	14.9	15.8	39.6	29.7	100	
Total	19.3	28.1	36.6	16	100	
Respondents’ Classification	Sector of Work					
	Private	Academic	Government		Total	
GDP	67 (72.9)	2.3 (2.1)	30.7 (25)		100 (60)	
Endodontist	34 (35.5)	18.6 (19.4)	47.4 (45.2)		100 (96.9)	
Endo Postgrad	0 (0)	0 (0)	100 (100)		100 (100)	
Other	9.1 (16.7)	36.4 (0)	54.5 (83.3)		100 (80)	
Total	57.6 (70)	8.1 (87.5)	38.2 (71.9)		100 (71.9)	

The values in parentheses represent proportion of respondents who used NiTi-RIs

Whilst most GDs (67%) worked in *private sector*, the highest proportion of endodontists (47.4%) (excluding residents and students in endodontic postgraduate programmes) worked in *governmental sector* (*p < 0.001).* Overall, there were no correlation between work’ sector and *NiTi-RIs usage* (*p = 0.183)* (Table 2).

Significantly, most respondents (62.5%) had been using *NiTi-RIs* for *More than 3 years (p < 0001)*; with significantly more endodontists (77.4%) than GDs (54.27%) *[p < 0.001]* (Table 3). As the number of *weekly performed RCTs* increased, the *experience in using NiTi-RIs* increased (*p < 0.001*).

**Table 3:** Experience Period in Using NiTi-RIs

	Duration of NiTi-RIs Usage					Total
	Up to 1 month	6 months	One year	3 years	More than 3 years	
Respondents’ Classification						
GDPs	4.2	4.2	28.9	16.7	54.2	100
Endodontists	0	0	0	22	77.4	100
Endo Postgraduate students	0	0	41.7	16.7	41.7	100
Others	0	8.3	16.7	8.3	66.7	100
Total	2.3	2.7	14.2	18.4	62.5	100
RCTs Cases Performed Per Week						
1–3	0	6.7	33.3	20	40	100
4–6	10	4.4	11.8	29.4	45.6	100
7–12	0	0	11.7	18.4	69.9	100
More Than 12 Cases	0	2.2	4.4	0	93.3	100
Total	2.3	2.7	14.2	18.4	62.5	100

Significantly, the highest proportion of respondents (45.3%) used NiTi-RIs because of *faster root-canal preparation (p < 0001)* (Table 4). The proportion of endodontists who were using RIs because of *keeping the original canal shape* (36.5%) was greater than that of GDs (15.2%) *[p < 0.001]*. Within the non-user of NiTi-RIs, there was no significant difference, regarding the *reasons for not using NiTi-RIs*, between those who *tried them before* (51%) and those who *did not* (49%)*, (p = 0.843)*. The majority (85.3%) did not use RIs because of *high-cost (p < 0.001)* (Table 4)*.*

**Table 4:** Reasons for Using/Not Using NiTi-RIs

Participants’ Classification	Reasons for Adopting RIs						
	Faster preparation	Keep canals’ original shape	Cutting efficiency	Less operator fatigue	Flexible	Others	Total
General Dentists	45.7	15.2	8.7	6.5	10.9	5.9	100
Endodontists	44.2	36.5	8.7	9.6	1	0	100
Others	50	25	8.3	8.3	8.4	0	100
Total	45.3	24.4	8.7	7.9	6.7	7.1	100
Previous experience	Reasons for not Adopting RIs						
	Difficult use	No cutting efficiency	Prone to fracture	No advantages	High Cost	Total	
Total	5.9	2.9	3.9	2	85.3	100	

The highest proportion (43.3%) reported *better undergraduate education* as the most important factor that can significantly increase NiTI-RIs usage in Saudi dental practice (*p < 0.001)* (Table 5). Overall there were no significant differences between endodontists and GDs *(p = 0.170)*.

**Table 5:** Increase of Using NiTi-RIs in Dental Practice

Respondents Classification	Factors Contributing into Increase of NiTi-RIs Usage					Total
	Lower cost	Better undergraduate education	Better post-graduation education	Higher treatment fees	Strict legalizations	
General Dentists	29.9	40.3	13	7.8	9.1	100 (231)
Endodontists	25.6	53.5	16.3	4.7	0	100 (43)
Others	6.7	60	33.3	0	0	100 (15)
Total	28	43.3	14.5	6.9	7.3	100 (289)

### Modalities of NiTi-RIs usage

#### Glide-path preparation

The majority (91.8%) used to prepare *glide-path (p < 0.001*); most of them (63.3%) were doing sot using *SS Hand-files (p < 0.001)* (Table 6). The proportion of endodontists who used SS Hand-files (75%) was significantly greater than that of GDs (55.8%) *[p = 0.011]*.

**Table 6:** Modalities of using NiTi-RIs (Preparation of Glide Path before using NiTi-RIs, Preparation Sequences and Implimentation of Hybrid Instrumentation Technique)

Respondents’ classification	Patterns of Glide-Path Preparation before using NiTi-RIs						Total
	Don’t Prepare	Yes Prepare Glide-Path using:					
		SS Hand-files	NiTI Hand-files		NiTi & SS Hand-files		
General Dentists	10.4	50	10.4		29.2		100
Endodontists	8.6	68.6	2.9		20		100
Others	0	50	33.3		16.7		100
Total	9.2	57.5	8.4		24.9		100
		91.8					
		63.3	9.3		27.4		
Respondents’ Classification	Preparation Sequence						Total
	Whole Canal	Rotary Coronally and Hand-files (NiTi or SS) Apically	Hand-files Coronally & Rotary Apically		GG burs Coronally and Rotary Apically		
General Dentists	57.4	8.5	12.8		21.3		100
Endodontists	64.5	6.5	3.2		25.8		100
Others	41.7	16.7	0		41.7		100
Total	57.8	8.1	8.1		26		100
Respondents’ classification	Pattern of Using Hybrid Technique						Total
	Always	Generally	Frequently	Sometimes	Rarely	Never	
General Dentists	8.3	10.4	8.3	33.3	20.8	18.8	100
Endodontists	18.1	10.5	9.5	18.1	25.7	18.1	100
Others	16.7	16.7	33.3	16.7	16.7	0	100
Total	12.6	10.7	10	26.4	22.6	17.6	100
	82.4						

#### Preparation sequence

Significantly, most participants (57.8%) used RIs for preparing the *Whole root-canal system* (*p < 0.001*), with significantly more endodontists (64.5%) than GDs (57.4%) *[p = 0.032]* (Table 6). Also, the proportion of endodontists who used gates glidden (GG) drills for preparing the coronal portion first (30.8%) was significantly greater than that of GDs (25.3%).

#### Using of hybrid technique

The majority (82.4%) used the *hybrid technique* (using different NiTi-RIs for instrumentation of one root-canal system) at least once *(p < 0.001*) (Table 6). Most of them (26.4%) used this technique *Sometimes (p < 0.001)*.

#### Using NiTi-RIs according to teeth types & canals’ geometrise

Overall, the majority (86%) used NiTi-RIs in *all teeth* with no significant differences between endodontists and GDs *(p = 0.092)* (Table 7). Significantly, most respondents (55.8%) used *NiTi-RIs* in all types of *canal curvatures* followed by those who used RIs in *straight and moderately curved* canals (34.5%) *[p < 0.001]*.

**Table 7:** NiTi-RIs usage according to teeth types & canals’ geometries

Respondents	NiTi-RIs Use According to Teeth’ Types								Total
	All teeth		Anterior		Anteriors & Molars		Premolars & Molars		
Total	86		0.4		2.3		11.2		100
NiTi-RIs Use According to Canals’ Curvature									
	All canals		Straight Canals	Moderately Curved	Straight & Moderately curved	Severely Curved	Moderately & Severely Curved		Total
Total	55.8		1.6	2.3	34.5	1.2	4.7		100
Respondents’ Classification	NiTi-RIs Use According to Canals’ Size			Total	NiTi-RIs Use According to Canals’ Shape				Total
	All	Narrow	Wide		All	Round	Round & Oval	Oval & C shaped	
General Dentists	75.6	13.3	11.1	100	85.1	4.3	8.5	2.1	100
Endodontists	89.9	10.1	0	100	78.1	11.4	10.5	0	100
Total	82.1	11.4	6.5	100	81.4	7.4	10.1	1.2	100

The majority of respondents (82.1%) used RIs in *all canals (regardless their sizes)*; with a significantly greater proportion of endodontists (89.9%) compared to GDs (75.6%) *[p = 0.003]* (Table 7). Also, the majority (81.4%) used RIs in *all canals shapes (p < 0.001)*.

## Discussion

Cleaning and shaping of the root-canal system is one critical step towards successful RCTs. In the last 30 years, considerable improvement has been witnessed in manufacturing instruments, especially NiTi-RIs, for safer and more predictable root-canal instrumentation [16]. Cleaning and shaping of the root-canal system using NiTi-RIs have been reported to be superior when compared SSIs [6, 8–10]. One previous study reported a significantly better success rate for teeth prepared with NiTi hand files than with SS K-files [6]. A very recent study has found that teeth instrumented with NiTi-RIs had a significantly better outcome when compared to SSIs [10]. Moreover, clinicians, recommend NiTi instruments for safer and faster instrumentation of the root-canal systems. Although several studies investigated the implementation of NiTi-RIs in dental practice of different countries, there is still lack of information on usage modalities and factors affecting dentists’ and specialists’ preferences on using them. This is especially true as each dental community has its own characteristics and influencing factors.

The current study showed that almost 72% respondents were using NiTi-RIs. As expected, more endodontists (96.9%) were using RIs compared to GDs (60%), which was in agreement with previous studies [12, 19]. Using of NiTi-RIs during endodontic postgraduate programmes’ training is a routine policy. In addition, endodontists are well aware of the advantages of using NiTi-RIs over SSIs such as: greater resistance to failure [4], greater centring ability [19, 20], less instrumentation time [21], less complications [9] and may lead to better outcomes [6, 22]. By contrast, the proportion of GDs who were using NiTi-RIs (60%) was significantly less than that of endodontists. These findings, however, show significant improvement in integrating NiTi-RIs in Saudi general dental practice, considering that only 3 and 17.5% of GDs had used them as reported in two studies published in 2010 [23] and 2014 [24]. Nonetheless, it should be noted that while the latter study was related to RCTs performed on molar teeth, the current study concerned using NiTi-RIs in all teeth. In addition, these figures could have been greater, taking into consideration the results of previous studies which were conducted many years ago; Switzerland (58%) [11], Australia (22%) [12], United Kingdom (65%) [13], Iran (50.6%) [19] and Wales (67%) [25]. One possible reason for this disagreement, as it will be discussed later, is the differences in undergraduate curricula. Also, postgraduate education, especially hands-on courses, could be another reason [19, 25–27]. Nevertheless, other reasons were proposed as influencing factors, such as: type of practice, perceived risk of complications, clinicians’ experience and other factors [12, 13, 19, 25, 28].

Previous studies showed significant correlation between clinicians’ experience and adoption of NiTi-RIs [12, 19, 29, 30]. The current study was not exception and showed a significant increased trend of using NiTi-RIs within the more experienced clinicians’ groups. Moreover, most respondents (62.5%) had been using them for *More than 3 years*. Also, there was a strong positive correlation between the number of weekly performed RCTs and the experience in using NiTi-RIs; with the vast majority (93.3%) of those who were doing *more than 12 cases per week* had been using RIs for *more than 3 years*. Unal et al referred to the high cost which discouraged young practitioners to use NiTi-RIs [30]. Parashos & Messer admitted that it is difficult to explain this greater preference among more experienced dentists [12]. However, they speculated that NiTi-RIs are unavailable for younger dentists who usually work as assistant dentists for more senior colleagues [12]. They also added that younger dentists may focus on perfecting hand instrumentation before shifting to a more advanced technique [12]. These justifications could be applied, to extent, to the current study’s results. The increased number of weekly performed RCTs within more experienced dentists could be another reason. The current results showed that using NiTi-RIs significantly increased as the number of weekly RCTs increased. It also showed that as the dentists’ experience increases, the weekly performed RCTs significantly increases. Previous studies identified the correlation between the average teeth treated per week and adoption of NiTi-RIs [12, 13, 19, 26, 31]. Only Barbakow & Lutz did not find such a correlation, which could be attributed to the specific and solo type of NiTi-RIs (Light-Speed) which was concerned in the study [11].

With a greater number of cases performed per week, saving time by using NiTi-RIs is paramount, as they significantly reduce instrumentation times [21]. A very recent study found that dentists performed significantly greater number of RCTs when using NiTi-RIs compared to manual preparation [32]. The highest proportion of respondents (45.3%), in the current study, used NiTi-RIs because of *faster root-canal preparation*. Similarly, faster canal preparation was the most important reason reported by Parashos & Messer (80%) and Mozayeni et al (69%) [12, 19]. Though faster root-canal instrumentation is one desired advantage, it should not be overemphasized. Maintaining the original shape of the root-canal system is one of the main objectives that was established long time ago [2] and is still considered as a gold standard. This may explain the significantly greater proportion of endodontists, compared to GDs, who reported the centring ability of the root-canal system as the second most important reason for using NiTi-RIs. This was in consistent with the results reported by Parashos & Messer and Mozayeni et al; 73 and 59%, respectively [12, 19]. It is generally accepted that with better cantering ability, less procedural errors (zipping, ledge formation or apical perforation) are expected.

By contrast, results of previous studies were inconsistent regarding the reasons behind not using NiTi-RIs. The vast majority of dentists (85%), in the current study, did not use NiTi-RIs because of high cost. Also, most dentists in Wales (62%) reported high cost for not using RIs [32]. Such a factor may reflect the real willingness of these dentists to use NiTi-RIs. By contrast, while the cost was the third most important obstacle (20%) reported by Australian dentists, *no perceived advantages* (36%) and *too fragile* (24%) were the first and second most important disincentives [12]. Mozayeni et al showed that dentists did not use NiTi-RIs due to *lack of adequate education* (46.7%), *non-availability* (46.7%) and *no perceived advantages* (37.8%) [19]. The time-interval between the two latter studies and the current one (15 and 8 years, respectively) may be one main reason for such discrepancy. Also, different clinics’ settings-up and environments may be additional reasons. In a more recent study, there was a clear discrepancy in reasons reported by dentists who were working in two different environments and settings [33]. While 75% of those working in *hospital* dental clinics did not use NiTi-RIs due to *no perceived benefit*, 42% of those working in *community* dental clinics did so due to high cost. It is very clear in this study that the different types of practice, with different setting and funds, can affect dentists’ preferences on NiTI-RIs adopting. The guidelines in England and Wales recommend a single use of NiTi-RIs to minimize the risk of Creutzfeldt-Jakob disease transmission [34]. Such a measure, as authors explained, may pose a cost pressure on using NiTi-RIs in general dental practices of the National Health Service (NHS). Nevertheless, while this was apparent in the latter study, it may not be applied to other countries, with different regulations, environments and treatment guidelines as well as different economic circumstances. The results of our study did not find a correlation between the type of practice and dentists’ preferences on adopting NiTi-RIs. There was no significant difference between *private* and *government* sectors regarding the use of RIs. However, whilst most GDs (67%) worked in the *private sector*, most endodontists (47.4%) worked for the *government.* It is well accepted that government sector’s clinics, in Saudi Arabia, are well equipped compared to private clinics. This may be an additional reason why more endodontists (96.9%) were using NiTi-RIs compared to GDs (60%). However, this is also a justification to accept the relatively good adoption of NiTi-RIs by GDs. In the last few years, postgraduate educational courses have received significant attention among dentists in Saudi Arabia and in regional countries. This could have contributed into better awareness among dentists about the advantages of using NiTi-RIs. This was also reflected on participants’ responses regarding the measures and factors that, they believe, will significantly improve adoption of NiTi-RIs in Saudi dental practice. The highest proportion (43.3%) reported *better undergraduate education* as the most important factor. Interestingly, also there was no difference in this belief between those who were using NiTi-RIs and those who were not. Reit et al addressed the importance of education on NiTi-RIs uptake in a well-designed and soundly executed study [26]. The uptake increased from 4% before educational programmes to 73% after undergoing the programmes. Interestingly, lectures combined with hands-on training resulted in a significantly better NiTi-RIs adoption than only a lectures-based programme (94 and 43%, respectively). Another well-designed study revealed that 77% of GDs who undergone an endodontic educational programme were using NiTi-RIs compared to only 6% of those who did not enrolled in the educational programme [27]. More importantly, a follow up study showed that the educational programmes resulted in a significantly less number of sessions to complete RCTs [28]. Consequently, authors concluded that instrumentation performed after the educational programmes was more cost-effective [28]. This may explain the results of the current study as participants reported the *lower cost* of NiTi-RIs as the second most important measure that will increase their usage.

A glide-path preparation has been defined as a smooth passage from the orifice to the apical foramen [35]. It is a desired negotiation and enlargement of the original canal anatomy that establish access to the apical region of the root-canal creating a path for subsequent instruments to follow. Manufacturers usually suggested creating a glide-path up to a size #15 or #20 hand file, especially SSIs, at working length before using NiTi-RIs. Unfortunately, none of previous studies addressed this important aspect. The majority of respondents (91.8%), in the current study, used to prepare a glide-path. This reflects clinicians good awareness, especially endodontists, of the advantages that glide-path can secure. Glide-path preparation can reduce torsional stresses on NiTi-RIs, hence enhance their resistance to fracture [36, 37]. Also, it can reduce modification of the root-canal geometry after cleaning and shaping [38, 39]. Glide-path can be prepared by SS hand files, NiTi hand files, or NiTi rotary files [35, 40, 41]. The highest proportion of those who prepared glide-path before using NiTi-RIs (63.3%), in the current study, were doing so using SS Hand-files. The proportion of endodontists who used SS Hand-files (75%) was significantly greater than that of GDs (55.8%). Endodontists are more aware of the need of rigid files that will first negotiate root-canals that usually exhibit some micro-calcifications and constrictions. Nevertheless, different results have been reported regarding the best instruments for creating glide-path [38].

Significantly, most respondents (57.8%) used NiTi-RIs for preparing the *Whole root-canal system*. Most manufacturers of NiTi-RIs, if not all, produce NiTi-RIs as integral systems, which consists of opener shapers and files with different tapers for preparation of the whole root-canals. Nevertheless, our results are inconsistent with a previous study, in which most respondents (69.8%) used to flare the coronal part with gates-glidden (GG) drills then use NiTi-RIs for preparing the apical part [19]. Whilst, the second highest proportion of respondents (26%), in the current study, were using GG drills for the coronal part first, then they used NiTi-RIs for the apical portion. These findings are similar to the study by Parashos & Messer [12]. Pre-flaring of the coronal third of root-canals usually secures easier inserting of NiTi-RIs to the apical portion and reduces the incident of instruments fracture [36]. Endodontists, in the current study, showed relatively better awareness of such advantage compared to GDs. Also, further statistics showed that the trend of using GG drills first for flaring the coronal part of the canal then complete preparing using NiTi-RIs increased significantly with less experienced participants.

The concept of *hybrid instrumentation* includes the use of NiTi-RIs of different systems to manage individual clinical situations; to achieve the best biomechanical cleaning and shaping and the least procedural errors [42]. This can contribute, especially in difficult cases, into less instruments fracture [42], which may explain why the majority of respondents, in the current study, used this technique at least once. However, this technique usually is not needed in all cases, depending on each case, which also explains why the highest proportion of this technique users (26.4%) were doing so *Sometimes*. While manufacturers claim that NiTi-RIs can be used in all cases regardless of their difficulties, clinical experience has shown that some cases can’t be prepared completely with NiTi-RIs [43]. The main challenging cases usually include either those of severely curved canals or with strange cross-section shape. The main concerns in such cases is instruments fracture, inability to follow the severely curved canals and insufficient cleaning and shaping of root-canals [16, 44]. This explains why the second highest proportion in the current study (34.5%) used NiTi-RIs in *straight and moderately curved canals*. Also, the majority of- and most of clinicians (86 and 55%, respectively) used NiTi-RIs in *all teeth* and in *all types of canal curvatures*, respectively. In addition, the majority used them in all canals *sizes* and *shapes* (82.1 and 81.4%, respectively). These findings are inconsistent with those obtained by Mozayeni et al, in which only 49 and 45% of respondents used NiTi-RIs in anterior teeth and premolars, respectively [19]. Also, only 61% of Australian dentists used them in anterior teeth [12]. One possible reason is the different NiTi-RIs available in the time of conducting each study. While the latter two studies were conducted almost 8 and 14 years ago, participants of the current study took the advantages of the significant improvement in NiTi-RIs’ designs, mechanical properties, and the mode of rotation in the last 10 years [16]. Nevertheless, the low proportion of respondents who preferred using NiTi-RIs only in specific *canal shapes* and *sizes* reflects the fact that the ideal NiTi rotary instrument is yet to be manufactured [16].

Like most studies, the current one has limitations. The response rates obtained in the current study (GDs: 52.37% and endodontists: 55.74%) are lower than those reported in previous similar studies [12, 25]. However, web-based questionnaires, like the current one, usually have lower response rates compared to self-administrative questionnaires [45]. Also, the lowest level of non-response bias could be obtained with 43% response rate [46]. Moreover, it is well accepted that low response rates but with good random and systematic sampling methods are better than high response rates without randomizations [18]. The current study was conducted following two pilot studies and a systematically random sampling method. Finally, one of the most important measure to validate results of questionnaire studies is to compare, responses to questionnaires’ main questions, between those who responded after the first sent-out (early responses) and those who responded after reminders (late responses) [12, 13]. The current study results showed no significant differences between the proportion of early respondents and that of late respondents regarding NiTi-RIs use (70 and, 75%, respectively). Another limitation could be related to some aspects that were not included in this study, such as inspecting the most common used types of NiTi-RIs, especially those systems that consist of a single file (i.e. Reciproc, Reciproc Blue, WaveOne, WaveOne Gold, OneCurve, etc). The main aims of this study were to establish the extent to which NiTi-RIs are used, to identify factors affect their adoption, to explore how they were used and how to increase their usage among practitioners. However, the current study results can be the foundation to conduct a further survey that investigates factors affect dentists’ preferences on specific type(s) or brand(s) of NiTi-RIs. This is especially true with the fast growing recent advances in producing new single file systems.

## Conclusions

Within the limitations of the current study, it can be concluded that NiTI-RIs are relatively well adopted in Saudi dental practice. However, better education, especially during undergraduate training, and lower cost may increase their usage. Overall, clinicians, especially endodontists, showed good awareness of NiTI-RIs usage aspects which reflected on usage modalities.

## Abbreviations

GDs: General dentists
NiTi-RIs: Nickel titanium rotary instruments
RCTs: Root canal treatments
SSIs: Stain less steel instruments
